# Brief Mindfulness Intervention vs. Health Enhancement Program for Patients Undergoing Dialysis: A Randomized Controlled Trial

**DOI:** 10.3390/healthcare9060659

**Published:** 2021-06-01

**Authors:** Marouane Nassim, Haley Park, Elena Dikaios, Angela Potes, Sasha Elbaz, Clare Mc Veigh, Mark Lipman, Marta Novak, Emilie Trinh, Ahsan Alam, Rita S. Suri, Zoe Thomas, Susana Torres-Platas, Akshya Vasudev, Neeti Sasi, Maryse Gautier, Istvan Mucsi, Helen Noble, Soham Rej

**Affiliations:** 1 McGill Meditation and Mind-Body Medicine Research Clinic (MMMM-RC) and Geri-PARTy Research Group, Jewish General Hospital, Montreal, QC H3T 1E2, Canada; hyeon.park3@mail.mcgill.ca (H.P.); elena.dikaios@mail.mcgill.ca (E.D.); angela.potesholguin@mail.mcgill.ca (A.P.); s_elba@live.concordia.ca (S.E.); zoe.thomas@mcgill.ca (Z.T.); gabriela.torresplatas@mail.mcgill.ca (S.T.-P.); neeti.sasi@mail.mcgill.ca (N.S.); maryse.gautier@mail.mcgill.ca (M.G.); soham.rej@mcgill.ca (S.R.); 2 Department of Psychiatry, McGill University, Montreal, QC H3A 0G4, Canada; 3 School of Nursing and Midwifery, Queen’s University, Belfast BT7 1NN, UK; clare.mcveigh@qub.ac.uk (C.M.V.); helen.noble@qub.ac.uk (H.N.); 4 Division of Nephrology, Jewish General Hospital, Montreal, QC H3T 1E2, Canada; mark.lipman@mcgill.ca; 5 Research Institute of the McGill University Health Center, McGill University, Montreal, QC H3A 0G4, Canada; emilie.trinh@mcgill.ca (E.T.); ahsan.alam@mcgill.ca (A.A.); rita.suri@mcgill.ca (R.S.S.); 6 Department of Psychiatry, University Health Network, University of Toronto, Toronto, ON M5S 1A1, Canada; marta@nefros.net; 7 Division of Nephrology, McGill University Health Centre, Montreal, QC H3A 0G4, Canada; Istvan.mucsi@uhn.ca; 8 Centre de Recherche du Centre Hospitalier de l’Université de Montréal, Montreal, QC H2X 0A9, Canada; 9 Geriatric Mood Disorders Lab, Department of Psychiatry, Western University, London N6A 3K7, UK; akshya.vasudev@lhsc.on.ca; 10 Transplant Inpatient Unit, Division of Nephrology, University Health Network, Toronto, ON M5G 2C4, Canada; 11 Department of Medicine, Faculty of Medicine, University of Toronto, Toronto, ON M5S 1A1, Canada

**Keywords:** meditation, mindfulness, depression, anxiety, dialysis

## Abstract

Background: Between 20–50% of patients undergoing maintenance dialysis for end-stage kidney disease experience symptoms of depression and/or anxiety, associated with increased mortality, greater health care utilization, and decreased quality of life. It is unknown whether mindfulness-based interventions can improve depression and anxiety symptoms in patients receiving this treatment. Methods: We conducted an 8-week multicenter randomized controlled trial comparing a brief mindfulness intervention (BMI) vs. an active control (Health Enhancement Program [HEP]) in 55 patients receiving dialysis with symptoms of depression and/or anxiety. The primary outcome was change in Patient Health Questionnaire-9 (PHQ-9) depression scores, with a primary analysis in participants with baseline PHQ-9 ≥ 10, and a secondary analysis including all participants. The secondary outcome was change in Generalized Anxiety Disorder-7 (GAD-7) anxiety scores with corresponding primary and secondary analyses. Results: Both BMI and HEP reduced depressive symptoms, with no difference between trial arms (PHQ-9 change = −7.0 vs. −6.1, *p* = 0.62). BMI was more effective than HEP in reducing anxiety (GAD-7 change = −8.7 vs. −1.4, *p* = 0.01). Secondary analyses revealed no differences between arms. Conclusions: For patients undergoing dialysis, both BMI and HEP may be helpful interventions for depression symptoms, and BMI may be superior to HEP for anxiety symptoms. Mindfulness-based and other psychosocial interventions may be further evaluated in those undergoing dialysis as treatment options for symptoms of depression and anxiety.

## 1. Introduction

Over 500,000 Americans with end-stage kidney disease (ESKD) receive maintenance dialysis each year [1]. Up to 50% of patients undergoing dialysis experience symptoms of depression and anxiety, while 20% of patients meet formal criteria for depressive and anxiety disorders [2,3,4]. Depression is characterized by having symptoms such as persistent low affect, lack of enjoyment in previously enjoyed activities, insomnia or hypersomnia, changes in appetite, low energy, psychomotor slowing, guilt, as well as feelings of helplessness and hopelessness [2,3,4]. Symptoms of depression and anxiety are associated with increased mortality [5,6], two-fold increased hospitalization rates [7] increased dialysis nonadherence [8], and reduced quality of life [8,9]. However, levels of depression and anxiety are rarely assessed in patients receiving dialysis, and the majority of affected patients are not receiving effective treatment [10,11]. The lack of systematic assessments may partially be attributed to limitations of current treatments. Evidence for the effectiveness of antidepressants in patients undergoing dialysis with depression and anxiety symptoms is low [12] and a recent large randomized controlled trial (RCT) found antidepressant therapy to be no better than placebo in non-dialysis-dependent patients with chronic kidney disease (CKD) [13]. Concerns related to polypharmacy, as well as increased risk of toxicity due to reduced renal clearance, may also limit use of pharmacotherapy in dialysis patients [14].

Mindfulness-based interventions, which involve the cultivation of non-judgmental, present-centered awareness, effectively reduce psychological symptoms in patients with chronic physical health problems [15,16]. Mindfulness meditation originates from Buddhist/Eastern origins and teaches practitioners how to be aware of and non-judgmental toward the present moment, which can translate into improved emotional, mental, and physical well-being. Mindfulness-based interventions incorporate these practices into structured therapeutic programs which are promising in terms of patient acceptability and scalability [17]. To date, the majority of research has focused on patients with cancer diagnoses [18,19]. A pilot study by our group found that brief chair-side mindfulness meditation was feasible, enjoyable, and could significantly reduce depressive symptoms in a subgroup of patients undergoing dialysis with greater baseline depression symptom burden [20]. Previous studies with mindfulness components in the ESKD and dialysis population have evaluated mindfulness-based stress reduction (MBSR), meditation techniques such as Benson’s technique and cognitive behavioral therapy (CBT), and positive psychology with components of mindfulness. Varying results, albeit with a general trend toward improvement in measures including depression, anxiety, stress, sleep, and quality of life, have been reported [21,22,23,24,25,26]. However, existing studies remain limited in number and suffer from a lack of active control comparators. Therefore, in order to address this gap in the literature, we aimed to conduct a randomized controlled trial (RCT) to evaluate the efficacy of a brief mindfulness intervention (BMI) against an active control health enhancement program (HEP) in reducing symptoms of depression and anxiety in patients undergoing dialysis. We hypothesized that BMI would be more effective than HEP in reducing symptoms of depression and anxiety. We also aimed to assess feasibility and participant experience for future scaling up of the intervention.

## 2. Methods

### 2.1. Study Design

We conducted an 8-week assessor-blinded parallel RCT comparing BMI vs. HEP. Prior to recruitment, the trial had been registered (ClinicalTrials.gov Identifier: NCT03406845). The study was approved by the research ethics boards at all participating hospitals. 

### 2.2. Participants

Participants were recruited and enrolled by three research assistants between May 2018 and March 2019 from in-center dialysis units at four tertiary-care hospitals in Montreal, Canada: the Jewish General Hospital, Centre hospitalier de l’Université de Montréal (CHUM), and the McGill University Health Centre (Montreal General Hospital and Lachine Hospital). Adult participants aged ≥18 years were included if they were receiving in-center thrice weekly dialysis for any duration and had symptoms of depression and/or anxiety, as indicated by a score ≥ 6 on the Patient Health Questionnaire (PHQ-9) [27] and/or the General Anxiety Disorder-7 (GAD-7) [28] Scores > 5 on these scales are associated with lower quality of life, and more disability days and primary care visits [27,28]. Patients were excluded if they had significant cognitive impairment suggestive of dementia (score < 3 on the Mini-Cog) [29], showed signs of acute psychosis, were experiencing suicidal ideation or intent as assessed by item 9 of PHQ-9, were currently receiving psychotherapy, were an incident patient, had hearing difficulties, or did not speak English or French. All participants gave informed written consent. 

### 2.3. Randomization and Methods to Reduce Bias

Participants were allocated in a 1:1 ratio to BMI or HEP. An independent statistician performed randomization using a computerized random number generator. Randomization was stratified by site and baseline PHQ-9 score (≥10 vs. <10). Stratification by baseline scores allowed for subgroup analyses of more severely depressed participants who might show greater treatment response, as suggested from our previous pilot research that found significant reduction in depressive and anxious symptoms in participants with a previous or current mental health diagnosis [20]. A PHQ-9 score ≥ 10 has a sensitivity of 88% and a specificity of 85% for major depressive disorder in the general population and indicates moderate to severe symptoms [30]. The independent statistician directly transmitted participant group information to the interventionists. Assessors were blinded to participant group assignment and the study was advertised as the investigation of two alternative treatments to depression and anxiety in dialysis patients to reduce expectancy bias in participants and referring clinicians.

### 2.4. Sample Size

Based on our previous feasibility trial of brief chair-side mindfulness in patients undergoing dialysis [20], we observed another effect size of 0.23 on depressive symptoms, which at two-tailed alpha = 0.05 and power 80% could require a sample size of 143 to demonstrate a statistical signal. However, based on mindfulness-based intervention studies in similar depressed samples with chronic severe physical illness, including an active comparator group controlling for clinical attention and social support elements of the intervention, a sample size of 175–300 may be needed [31,32]. For this reason, we aimed to recruit 30 participants in each arm for this pilot trial, standard for an initial RCT comparing the intervention with an active control, to generate a sample size estimate for a definitive RCT [33].

### 2.5. Treatment: Brief Mindfulness Intervention (BMI)

While undergoing dialysis, participants received two chair-side 20 min sessions of BMI per week for 8 weeks. Most sessions included around 15 min of guided mindfulness meditation techniques drawn from mindfulness-based cognitive therapy (MBCT) [34]. These mindfulness meditation techniques included a body scan, mindful eating, guided breath meditation, mindful movement, and loving-kindness meditation. Techniques emphasized paying attention to specific elements of one’s moment-to-moment sensory experience with a non-judgmental attitude. In addition, participants learned material about mindfulness and how to apply it to daily life. Participants could individually check-in with the interventionist and ask any questions for 3–5 min after each session. Interventions were delivered in English or French via audio headsets, allowing up to 4–6 participants to receive instruction simultaneously while an interventionist delivered instructions in patients’ sightline. A 10-minute daily home mindfulness practice was encouraged. 

### 2.6. Active Control: Health Enhancement Program (HEP)

HEP was previously designed and used as an active control in mindfulness-based intervention trials to control for several non-program-specific intervention factors including facilitator attention, expectation for positive change, treatment duration, format (e.g., individual vs. group), and time spent on at-home practice [35,36]. It was structurally equivalent to the mindfulness meditation program (two 20-minute sessions per week for 8-weeks, delivered via audio-headsets in groups of 4–6 participants, with 3–5 min for questions or discussion), and encouraged the same amount of home practice (implementing health-enhancing habits for 10 min daily). Each session involved educational and activity-based sessions on light exercise, sleep, stress and anxiety, nutrition, journaling, and music enjoyment with drawing.

### 2.7. Interventionists

Two interventionists delivered the interventions at all four sites. One interventionist was a registered social worker with facilitator certification in MBCT and a personal mindfulness practice of over 7 years. The other was a psychologist and certified MBSR teacher and a MBCT facilitator with over 40 years of clinical mental health experience. Both the intervention and active control programs were delivered and reviewed by the same interventionist at any given site to control for the effect of interventionist characteristics and ensure consistency.

### 2.8. Feasibility Outcome Measures

We aimed for a recruitment goal of 60 participants within 18 months, a drop-out rate of less than 30%, and a non-attendance rate of less than 30% of participants (excluding dropouts) failing to attend 75% of their assigned intervention (12/16 sessions). 

### 2.9. Efficacy Outcome Measures

The primary outcome was the between-group difference in the 8-week change in depressive symptoms as measured by the PHQ-9, with a prespecified primary analysis in the subgroup of patients with baseline PHQ-9 scores ≥ 10, and a secondary analysis with all randomized participants. The PHQ-9 is a widely-used 9-item self-report questionnaire used to assess depressive symptom severity. Scores for each item range from 0 (not at all) to 3 (nearly every day). Total scores can range from 0–27. The scale has good internal consistency (Cronbach’s α = 0.89) and test-retest reliability (intraclass correlation = 0.87) [27].

The secondary outcome was the between group difference in 8-week change in anxiety symptoms as measured by the GAD-7, with a prespecified primary analysis in the subgroup of patients with a baseline GAD-7 score ≥ 10, and a secondary analysis in all randomized participants. The GAD-7 is a 7-item scale measuring symptoms of anxiety, with scores for each item ranging from 0 (not at all) to 3 (nearly every day). Total scores can range from 0–21. The scale has good internal consistency (Cronbach’s α = 0.92) and test-retest reliability (intraclass correlation = 0.83) [28].

### 2.10. Qualitative Evaluation

Upon completion of the trial, we developed a 10-item survey with open-ended questions to collect feedback from participants who completed 8 weeks of BMI (*n* = 12) or HEP (*n* = 17) at a single dialysis unit. The survey asked questions regarding participants’ perceived benefit, skills learned, and satisfaction with the program.

### 2.11. Analyses

Normality of the data was assessed with the Shapiro–Wilk test. Baseline characteristics were compared between arms using independent *t*, Mann-Whitney *U*, or chi-square tests. We analyzed primary and secondary outcome measures on difference scores from baseline to 8 weeks between trial arms using independent *t* tests, with the exception of the primary outcome secondary analysis, where a Mann–Whitney *U* test was used. Analysis of differences between pre-to-post scores within trial arms were conducted using paired *t* tests or Wilcoxon signed rank tests. Measures of effect size at 95% confidence intervals were conducted using Cohen’s *d* statistic or *r* (Z/√N). Effect sizes were interpreted as follows; *d*: small effect = 0.2, medium effect = 0.5, large effect = 0.8; *r*: small effect = 0.1, medium effect = 0.3, large effect = 0.5 [37]. Participants with missing data were reported but excluded from the outcome analyses. Qualitative data from participant feedback were analyzed using inductive thematic analysis to identify overarching themes that emerged from the data [38,39].

## 3. Results

### 3.1. Participant Flow

**Figure 1:**
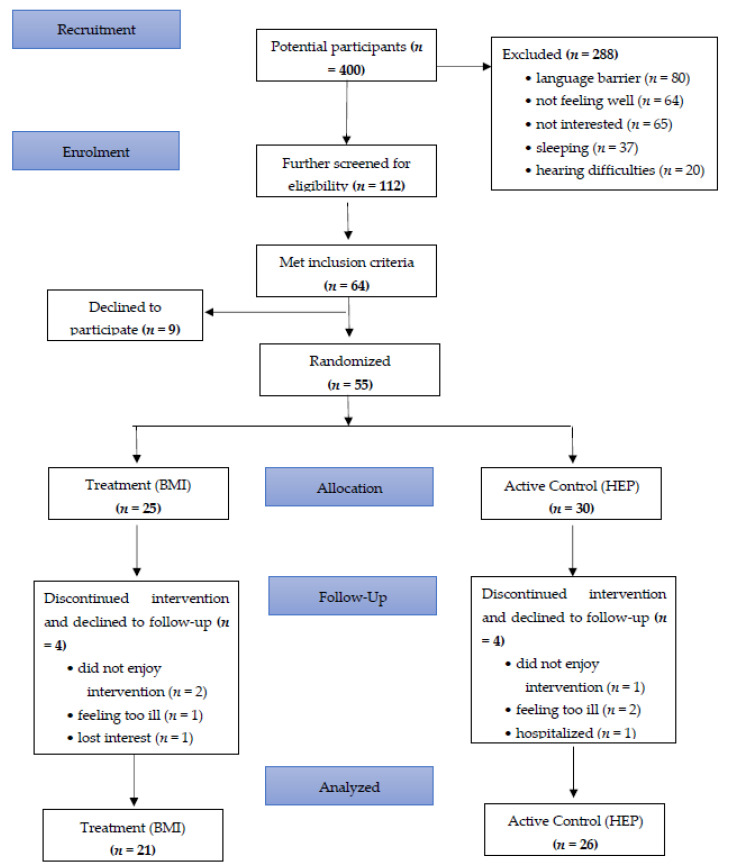
Participant flow diagram.

Of 400 potential participants at the four dialysis units, 112 were screened with the PHQ-9 and GAD-7. They were also screened for eligibility, and 64 met eligibility criteria (Figure 1). Of these, 9 were refused and 55 were randomized. Four participants in each arm dropped out of the study and declined follow-up assessments. Some of the most common reasons for participant attrition were due to feeling too ill, too distressed to participate, or not enjoying the interventions. In total, 21 of 25 participants in the treatment arm and 26 of 30 participants in the active control arm completed the intervention and follow-up assessments. 

### 3.2. Baseline Characteristics

Baseline clinical and demographic characteristics between the treatment and control groups are presented in Table 1. Baseline characteristics did not significantly differ between the two arms. Participants had a similar number of medical conditions (7.0 (IQR 5.0–8.0)) and were taking an average of 10.0 (IQR 8.0–13.0) medications. Twenty-nine percent of participants had a history of a psychiatric diagnosis, including depression (18.2%), and anxiety (7.3%); 7.3% were receiving mental health follow-up at the time of the study. 

### 3.3. Feasibility

A total of 55 participants were recruited for the study within 18 months. Eight participants dropped out of the study (4 treatment, 4 control) and did not complete post-assessments, leading to a drop-out rate of 14.5% (16.0% treatment, 13.3% control). Of all participants, 10 failed to attend 75% of the intervention (4 treatment, 6 control), leading to a non-attendance rate of 21.3% (19.0% treatment, 23.1% control). 

### 3.4. Efficacy Outcomes

Primary analyses of the primary (depression) and secondary (anxiety) outcomes included participant subgroups with baseline PHQ-9 or GAD-7 scores ≥ 10, respectively (Table 2). There was no significant difference in PHQ-9 change from baseline to 8 weeks between groups (BMI −7.0 pts (±2.6) vs. HEP −6.1 pts (±4.4), 95% CI [−4.7, 2.9], *p* = 0.62, *d* = 0.3), but there was a significant difference in GAD-7 change (BMI −8.7 pts (±2.1) vs. HEP −1.4 pts (±5.4), 95% CI [−12.6, −1.9], *p* = 0.01, *d* = 1.8).

Secondary analyses of the primary (depression) and secondary (anxiety) outcomes included all participants who completed the study, regardless of baseline PHQ-9 or GAD-7 scores (Table 3). There was no significant difference in PHQ-9 change from baseline to 8-weeks between groups (BMI −5.0 pts (IQR = −7.0–−1.0) vs. HEP −3.0 pts (IQR = −5.0–0.0), 95% CI [−4.0, 1.0], *p* = 0.23, *r* = 0.2), or in GAD-7 change (−3.1 pts (±4.7) vs. −1.0 pt (±3.9), 95% CI [−4.6, 0.4], *p* = 0.10, *d* = 0.5). 

Further exploratory analyses were conducted to test for significant differences in PHQ-9 and GAD-7 scores from baseline to 8 weeks within each trial arm. Both trial arms had significant reductions in PHQ-9 scores in the subgroup of those with higher baseline symptoms (BMI *p* ≤ 0.001, *d* = 2.7; HEP *p* ≤ 0.01, *d* = 1.4) as well as in all participants (BMI *p* ≤ 0.001, *r* = 0.4; HEP *p* ≤ 0.01, *r* = 0.4). For change in GAD-7, BMI had significant reductions both in the subgroup of those with higher baseline symptoms and in all participants (*p* ≤ 0.001, *d* = 4.2; *p* = 0.01, *r* = 0.6), and HEP did not (*p* = 0.78, *d* = 0.3; *p* = 0.26, *r* = 0.2).

### 3.5. Qualitative Evaluation

The qualitative evaluation led to identification of themes that arose for participants of BMI (Table S1) including improved bio-psychosocial well-being, impact of interventionist support, non-engagement in homework, benefit from informal mindfulness practice, and challenges in practicing in the dialysis unit. Themes that arose for participants of HEP (Table S2) included improved bio-psychosocial well-being, impact of interventionist support, non-engagement in homework, practice becoming a part of daily life, desire for more information on program aspects, and barriers to physical exercise components.

## 4. Discussion

BMI was not more effective than the active control (HEP) in reducing symptoms of depression, although both interventions were feasible and associated with significant reductions in depressive symptoms from baseline. However, BMI was more effective than HEP in reducing symptoms of anxiety in the subgroup of more severely anxious participants at baseline. In addition, BMI was associated with significant reductions in symptoms of anxiety from baseline to 8 weeks, while HEP was not when considering all participants with either mild or moderate-to-severe symptoms. To summarize, both BMI and HEP appear to be efficacious in reducing symptoms of depression, and BMI but not HEP, to be efficacious in reducing symptoms of anxiety.

The finding that both BMI and HEP reduced depressive symptoms is consistent with previous studies examining mindfulness-based and other psychosocial interventions in the dialysis population. MBSR, CBT, and positive psychological interventions with mindfulness components observed reductions in mean depression scores from moderate-to-severe to mild levels [23,25,26,40]. A review of psychosocial interventions for patients receiving dialysis estimates a comparable effect size of these interventions for depression (Hodges = 0.44, medium) [32] to those found in our exploratory PHQ-9 pre-post within-group analyses. HEP’s efficacy in reducing depressive symptoms in the present study may be due to it being a strong active control, perhaps more similar to a structured psychoeducation program with effect magnitudes in the order of psychotherapies such as CBT [35]. Its therapeutically valid elements (e.g. healthy eating, light exercise, music/art therapy) likely contributed to its efficacy, in conjunction with non-specific factors such as facilitator attention. Our study also suggests that brief program formatting (400 total minutes), while about 40–70% shorter than most psychosocial interventions [23,25,26,40], may lead to similar benefits.

An interesting result of the study is in the improvement of anxiety in the BMI group and the absence of such improvement in HEP. This result may reflect anxiety being more responsive to specific techniques such as mindfulness or CBT. There is some evidence for this hypothesis in the dialysis literature: a psychoeducation intervention reduced symptoms of depression, but not anxiety [41]; and CBT was found to be superior to non-directive counseling in reducing anxiety [42]; There are fewer instances of evaluations of anxiety in psychosocial interventions in the dialysis population than depression, although it is associated with poorer health outcomes, independently of depression [43]; Anxiety is thus an outcome that can be targeted for future mindfulness-based interventions in this population. 

### 4.1. Strengths and Limitations

Strengths of this study include the use of a randomized controlled trial design and an active control comparator. Use of audio headsets and chairside delivery during dialysis procedures also allowed greater scalability and ease of access to treatment; reduced mobility, time, and energy present challenges in seeking and receiving mental healthcare. Limitations of the study include the facilitation of both BMI and HEP by the same interventionist at a study site, which, while controlling for interventionist characteristics, presented vulnerability to bias from interventionist allegiances. The necessity of specialist training for program delivery is a limitation in study and treatment implementation. Moreover, although we were aiming to recruit 60 participants, we were able to recruit 55 due to funding limitations, which nonetheless provides an adequate sample size to estimate efficacy of the interventions for future larger definitive studies. Moreover, although we initially wished to assess biomarkers in this study such as blood c-reactive protein (CRP), interleukin-6 (IL6), mature brain-derived neurotrophic factor (mBDNF), heart rate variability (HRV), and blood pressure (BP), due to funding and logistical challenges at the different hospitals, this was not pursued.

### 4.2. Future Directions

Future studies may leverage hybrid designs of online audio-visual delivery of interventions to significantly reduce interventionist burden and improve scalability, accessibility, and cost [44]. Virtual-delivery may be complemented with personal check-ins, which may be an important treatment factor for psychosocial interventions.

## 5. Conclusions

Both the Brief Mindfulness Intervention (BMI) and Health Enhancement Program (HEP) significantly reduced symptoms of depression in patients undergoing dialysis, but BMI was more efficacious than HEP in reducing symptoms of anxiety in individuals with greater baseline anxiety severity. Both BMI and HEP may thus be useful treatments in the in-center dialysis setting, although BMI may be more desirable given its efficacy for both symptoms of depression and anxiety. In light of the limitations of pharmacotherapy in patients undergoing dialysis, mindfulness and other psychosocial interventions present feasible, acceptable, and scalable treatment options for the large percentage of patients who experience depressive and anxious symptoms. Further research can establish their efficacy. 

## Figures and Tables

**Table 1:** Baseline Demographic and Clinical Characteristics.

Participant Data	Total Sample (*n* = 55)	Treatment Group (*n* = 25)	Active Control Group (*n* = 30)
Demographic information			
Female	58.2% (*n* = 32)	65.4% (*n* = 17)	51.7% (*n* = 15)
Age, yr	62.2 ± 12.0	58.7 ± 12.2	64.9 ± 11.2
Married/common law	38.2% (*n* = 21)	46.2% (*n* = 12)	30.0% (*n* = 9)
Level of education			
*Elementary School*	9.1% (*n* = 5)	8.0% (*n* = 2)	10.3% (*n* = 3)
*High school*	38.2% (*n* = 21)	30.1% (*n* = 8)	44.8% (*n* = 13)
*CEGEP (pre-university in Quebec)*	18.2% (*n* = 10)	23.1% (*n* = 6)	13.8% (*n* = 4)
*University*	29.1% (*n* = 16)	30.1% (*n* = 8)	27.6% (*n* = 8)
Currently living alone	38.2% (*n* = 21)	26.9% (*n* = 7)	48.3% (*n* = 14)
Medical History			
Number of medical problems	7.0 (IQR 5.0–8.0)	7.0 (IQR 5.0–8.0)	7.0 (IQR 5.0–9.0)
Number of years on dialysis	3.0 (IQR 1.0–7.0)	2.0 (IQR 1.0–5.0)	6.0 (IQR 1.0–9.0)
Number of current medications	10.0 (IQR 8.0–13.0)	9.0 (IQR 7.0–12.0)	11.0 (IQR 9.0–13.0)
**Mental health information**			
History of psychiatric diagnosis	29.1% (*n* = 16)	32.0% (*n* = 8)	26.7% (*n* = 8)
*Depression*	18.2% (*n* = 10)	24.0% (*n* = 6)	13.3% (*n* = 4)
*Anxiety*	7.3% (*n* = 4)	4.0% (*n* = 1)	10.0% (*n* = 3)
*Other*	3.6% (*n* = 2)	4.0% (*n* = 1)	3.3% (*n* = 1)
*Number of years since first diagnosis*	15.0 (IQR 3.0–20.0)	4.0 (IQR 3.0–15.0)	17.0 (IQR 1.0–35.0)
Psychotropic medications	30.1% (*n* = 17)	28.0% (*n* = 7)	33.3% (*n* = 10)
*Anti-depressant*	25.5% (*n* = 14)	28.0% (*n* = 7)	23.3% (*n* = 7)
*Anti-anxiety*	9.1% (*n* = 5)	4.0% (*n* = 1)	13.3% (*n* = 4)
Currently receiving mental health follow-up	7.3% (*n* = 4)	11.5% (*n* = 3)	3.4% (*n* = 1)
**Other**			
Habits (yes/no)			
*Smoking*	14.5% (*n* = 8)	19.2% (*n* = 5)	10.3% (*n* = 3)
*Alcohol consumption*	23.6% (*n* = 13)	15.4% (*n* = 4)	30.0% (*n* = 9)
*Recreational drugs*	16.4% (*n* = 9)	19.2% (*n* = 5)	13.8% (*n* = 4)
Meditated before	18.2% (*n* = 10)	23.1% (*n* = 6)	13.8% (*n* = 4)
Meditates currently	10.9% (*n* = 6)	11.5% (*n* = 3)	10.3% (*n* = 3)

**Table 2:** Primary analyses of primary and secondary outcomes: change in depression (PHQ-9) and anxiety (GAD-7) in participant subgroups (baseline PHQ-9 or GAD-7 ≥ 10).

**PHQ-9** **(*n* = 19)**	**Treatment (BMI)** **(*n* = 9)**	**Active Control (HEP)** **(*n* = 10)**
Baseline	12.8 (±4.2)	14.7 (±5.2)
Follow-up	5.8 (±3.3)	8.6 (±4.7)
8-week Change	−7.0 pts (±2.6)	−6.1 pts (±4.4)
Between-group Change	*p* = 0.62 95% CI [−4.7, 2.9]	
**GAD-7** **(*n* = 11)**	**Treatment** **(*n* = 6)**	**Active Control** **(*n* = 7)**
Baseline	13.8 (±3.3)	12.8 (±2.0)
Follow-up	5.2 (±4.6)	11.4 (±4.6)
8-week Change	−8.7 pts (±2.1)	−1.4 pts (±5.4)
Between-group Change	*p* = 0.01 * 95% CI [−12.6, −1.9]	

* significant differences.

**Table 3:** Secondary analyses of primary and secondary outcomes: change in depression (PHQ-9) and anxiety (GAD-7) from baseline to 8 weeks (*n* = 47).

**PHQ-9**	**Treatment (BMI)** **(*n* = 21)**	**Active Control (HEP)** **(*n* = 26)**
Baseline	9.3 (±3.2)	10.0 (±4.8)
Follow-up	5.2 (±3.3)	6.9 (±4.1)
8-week Change	−5 pts (−7–−1)	−3 pts (−5–0)
Between-group Change	*p* = 0.23 95% CI = [−4, 1]	
**GAD-7**	**Treatment (BMI)** **(*n* = 21)**	**Active Control (HEP)** **(*n* = 26)**
Baseline	7.0 (±5.4)	6.8 (±3.8)
Follow-up	3.9 (±4.0)	5.8 (±4.8)
8-week Change	−3.1 pts (±4.7)	−1 pts (±3.9)
Between-group Change	*p* = 0.1 95% CI = [−4.61, 0.4]	

## Data Availability

Individual participant data that underlie the results reported in this article, after deidentification, will be available immediately after publication with no end date, with investigators whose proposed use of the data has been approved by an independent review committee identified for this purpose. Proposals should be directed to soham.rej@mcgill.ca. To gain access, data requestors will need to sign a data access agreement.

## Supplementary Materials

The following are available online at https://www.mdpi.com/article/10.3390/healthcare9060659/s1, Supplementary Table S1: Treatment Group (MBI) Thematic Analysis; Supplementary Table S2: Active Control Group (HEP) Thematic Analysis.
