# Supplementary material for: Brief Mindfulness Intervention vs. Health Enhancement Program for Patients Undergoing Dialysis: A Randomized Controlled Trial
Source: Healthcare (Basel). 2021 Jun 1;9(6):659. doi: 10.3390/healthcare9060659 (PMC8228217; doi:10.3390/healthcare9060659)
Supplement: Supplementary file 1 [file healthcare-09-00659-s001.zip › healthcare-1237838-supplementary.pdf]

healthcare

MDPI

Supplemental Material

Table of Contents

1. Table S1: Treatment Group (BMI) Thematic Analysis

2. Table S2: Active Control Group (HEP) Thematic Analysis

Table S1. Treatment Group (BMI) Thematic Analysis.

Overarching Themes	Interpretative Themes	Descriptive Themes and Quotes
1. Therapeutic Benefits	Improved psychological well-being	**Improvements in participants' mood**<br>*"I've been able to control my anger much better."*<br>**Improved memory**<br>*"[Mindfulness] made me focus more and not on all my issues and problems, also helped me retain memory when reading."*<br>**Improved stress**<br>*"I used to be nervous and stressed much more [before the program]."*<br>*"[Mindfulness] helped to notice stress and get rid of bad thoughts."*<br>***Brings you closer to your soul***<br>*"It's [mindfulness] opening communication between my mind and body."*<br>*"It [mindfulness] brings you closer to your soul, puts you in the right direction instead of down or lost."*
	Improved physiological well-being	**Improved participants' blood pressure**<br>*"Morning meditation has helped me tremendously; as a matter of fact my blood pressure has gone down drastically."*<br>*"My blood pressure goes down because it [mindfulness] calms me."*
	Welcomed human interaction	**Enjoyed interaction with an instructor**<br>*"It was fun getting to know the instructor, too bad it has to come to an end."*
2. Practice and Homework	Didn't engage in homework	**Not enough time to do homework**<br>*"I am so busy and I didn't have time to do the meditation at home."*<br>**Home environment inhibited informal practice**<br>*"[Difficult to practice at home] because of my situation, family with 2 young kids, little sleep 3 – 4hrs; no space for quiet at home."*<br>**Did not like some of the informal practices**<br>*"I didn't enjoy the tapping exercise as I found it hard to get in to."*<br>***Too tired***<br>*"I like the [informal] mindfulness practices but I was often too tired to practice outside of sessions."*
	Informal mindfulness practices beneficial	**STOP (Stop, Take a breath, Observe, Proceed) exercise was helpful**

		"I think it's helpful. It makes you aware of your environment." *"I really enjoyed practicing this technique as it was easy and yielded a good result."* *"I practice [STOP exercise] when waiting when others are late, such as the nurse or [dialysis] transport, and it helps."* **Body Scan helps to engage with what is going on in the body** *"Body scan makes you get in touch with the body and you see what is happening."*
3. Challenges	Dialysis environment not optimal	**Interrupted during formal mindfulness practice** *"I was annoyed being interrupted in [mindfulness] session as not a closed room and had distractions."* ***Dialysis is stressful enough*** *"Situation [for conducting the program] wasn't right, somewhere else would have been better as dialysis is stressful enough."* **Disturbed during dialysis to practice mindfulness** *"I didn't like being woken up for [mindfulness] sessions."*
	Not enough information on mindfulness	**Perceived mindfulness to be a religious practice** *"I would have appreciated more background information about mindfulness because it felt a bit religious."* **Unclear of the purpose of mindfulness** *"Some of it [mindfulness] was very helpful but other parts less so because I was unclear of the goal [of the program]."*
	Increased symptoms	**Body scan caused physiological and psychological symptoms** *"I started to get itchy all over when doing the body scan."* *"I enjoyed the techniques taught by the instructor, except the body scan as it made me agitated."* *"I like the body scan but because I have constant pain it can amplify the pain."* **Formal practice cause hypotension during dialysis** *"[I experienced] lower blood pressure during sessions which in dialysis is not always good."*

Table S2. Active Control Group (HEP) Thematic Analysis.

Overarching Themes	Interpretative Themes	Descriptive Themes and Quotes
1. Therapeutic Benefits	Improved psychological well-being	**Improved Depression**<br>*"It [HEP] helped with depression, I try not to cry as much and have better mood."*<br>*"It [HEP] helped me to fight depression quite a bit and made me more aware."*<br>**Improved Mood**<br>*"[HEP] helps take me out of my suffering when I am in a bad mood."*<br>*"I used to yell at people at home but now I try not to."*<br>**Improved Sleep**<br>*"[I have been] sleeping better since I started [HEP]."*<br>*"Used it [HEP] when I had a hard time sleeping."*<br>***Broke Monotony***<br>*"Really enjoyed something [HEP] that broke up the monotony of being in dialysis."*
	Improved physiological well-being	**Increased physical activity**<br>*"I will keep pushing to exercise, and I am presently looking for a new hobby...dance class?"*<br>*"I feel better when exercising and I am taking longer walks now."*<br>**Gained knowledge of how to increase physical health through nutrition and exercise**<br>*"Health improvement [benefits]...it [HEP] put everything down in front of me of what I should be doing."*<br>*"Food section was beneficial, I learned a lot."*
	Welcoming of human interaction	**Face-to-face delivery enhanced program**<br>*"The program was well planned, well executed and the lady [instructor] took good care of me."*<br>*"She [instructor] makes it clear and reminds me what to do which makes things clearer."*<br>**Participants liked to have someone to talk to**<br>*"[HEP] was a good chance to talk, good opportunity to talk with someone else."*
2. Practice and Homework	Didn't engage in homework	**Other priorities**<br>*"It's [HEP homework] not a priority in my life."*<br>**Not enough time to do homework**<br>*"I was involved too much in hospital care so didn't have much time [for homework]."*<br>*"Lack of time, I am busier than I want to be."*
	Practice at home became part of everyday life	**Found ways to incorporate into daily activities**<br>*"I incorporated it into what I was already doing."*
3. Challenges	Not enough information	**Like to know more about stress management**<br>*"Prefer more emphasis on stress management."*<br>**More information needed on self-care**<br>*"More information on self-care, mental health concerns and mood and how these affect physical health*

Barriers to physical exercises	**Felt embarrassed carrying out exercises**<br>*"Sometimes I felt embarrassed as I can't move as well with exercise...self-conscious."*
